# Comparing microbiological and molecular diagnostic tools for the surveillance of anthrax

**DOI:** 10.1371/journal.pntd.0012122

**Published:** 2024-11-21

**Authors:** Sunday Ochonu Ochai, Ayesha Hassim, Edgar H. Dekker, Thuto Magome, Kgaugelo Edward Lekota, S. Marcus Makgabo, Lin-Mari de Klerk-Loris, Louis O. van Schalkwyk, Pauline L. Kamath, Wendy C. Turner, Henriette van Heerden

**Affiliations:** 1 Department of Veterinary Tropical Diseases, Faculty of Veterinary Science, University of Pretoria, Onderstepoort, South Africa; 2 Antimicrobial Research Unit, College of Health Sciences, University of KwaZulu-Natal, Durban, South Africa; 3 International Centre for Antimicrobial Resistance Solutions, Copenhagen S, 2300, Denmark; 4 Office of the State Veterinarian, Department of Agriculture, Forestry and Fisheries, Government of South Africa, Skukuza, South Africa; 5 Unit for Environmental Sciences and Management, Microbiology, North West University, Potchefstroom, South Africa; 6 Department of Life and Consumer Sciences, College of Agriculture and Environmental Sciences, University of South Africa (UNISA), Florida Campus, Roodepoort, 1709, South Africa; 7 Department of Migration, Max Planck Institute of Animal Behavior, Radolfzell, Germany; 8 School of Food and Agriculture, University of Maine, Orono, Maine, United States of America; 9 Maine Center for Genetics in the Environment, University of Maine, Orono, Maine, United States of America; 10 U.S. Geological Survey, Wisconsin Cooperative Wildlife Research Unit, Department of Forest and Wildlife Ecology, University of Wisconsin-Madison, Madison, Wisconsin, United States of America; Cornell University, UNITED STATES OF AMERICA

## Abstract

The diagnosis of anthrax, a zoonotic disease caused by *Bacillus anthracis* can be complicated by detection of closely related species. Conventional diagnosis of anthrax involves microscopy, culture identification of bacterial colonies and molecular detection. Genetic markers used are often virulence gene targets such as *B. anthracis *protective antigen (*pagA*, also called BAPA, occurring on plasmid pXO1), lethal factor (*lef*, on pXO1), capsule-encoding *capB/C* (located on pXO2) as well as chromosomal Ba-1. Combinations of genetic markers using real-time/quantitative polymerase chain reaction (qPCR) are used to confirm *B*. *anthracis* from culture but can also be used directly on diagnostic samples to avoid propagation and its associated biorisks and for faster identification. We investigated how the presence of closely related species could complicate anthrax diagnoses with and without culture to standardise the use of genetic markers using qPCR for accurate anthrax diagnosis. Using blood smears from 2012–2020 from wildlife mortalities (n = 1708) in Kruger National Park in South Africa where anthrax is endemic, we contrasted anthrax diagnostic results based on qPCR, microscopy, and culture. From smears, 113/1708 grew bacteria in culture, from which 506 isolates were obtained. Of these isolates, only 24.7% (125 isolates) were positive for *B*. *anthracis* based on genetic markers or microscopy. However, among these, merely 4/125 (3.2%) were confirmed *B*. *anthracis* isolates (based on morphology, microscopy, and sensitivity testing to penicillin and gamma-phage) from the blood smear, likely due to poor survival of spores on stored smears. This study identified *B*. *cereus sensu lato*, which included *B*. *cereus* and *B*. *anthracis*, *Peribacillus* spp., and *Priestia* spp. clusters using *gyrB* gene in selected bacterial isolates positive for *pagA* region using BAPA probe. Using qPCR on blood smears, 52.1% (890 samples) tested positive for *B*. *anthracis* based on one or a combination of genetic markers which included the 25 positive controls. Notably, the standard *lef* primer set displayed the lowest specificity and accuracy. The Ba-1+BAPA+*lef* combination showed 100% specificity, sensitivity, and accuracy. Various marker combinations, such as Ba-1+*capB*, BAPA+*capB*, Ba-1+BAPA+*capB*+*lef*, and BAPA+*lef*+*capB*, all demonstrated 100.0% specificity and 98.7% accuracy, while maintaining a sensitivity of 96.6%. Using Ba-1+BAPA+*lef*+*capB*, as well as Ba-1+BAPA+*lef* with molecular diagnosis accurately detects *B*. *anthracis* in the absence of bacterial culture. Systematically combining microscopy and molecular markers holds promise for notably reducing false positives. This significantly enhances the detection and surveillance of diseases like anthrax in southern Africa and beyond and reduces the need for propagation of the bacteria in culture.

## Authors summary

Our research tackles the challenges of diagnosing anthrax, a severe disease caused by the bacterium *Bacillus anthracis*, especially in regions where similar bacteria coexist. Traditional methods of identifying anthrax involve microscopic examination, bacterial culture, and genetic testing. We aimed to enhance the accuracy and speed of genetic testing to identify anthrax directly from samples, without the need for bacterial culture, thereby reducing the associated risks.

We analysed blood samples from wildlife deaths in Kruger National Park, South Africa, where anthrax is common. By applying advanced genetic tests, we found that over half of the samples tested positive for anthrax. We discovered that combining certain genetic markers significantly improved the accuracy of these tests, reaching up to 100% accuracy. This method helps reduce false positives and enhances the reliability of anthrax detection.

Our findings suggest that using a combination of genetic markers can accurately identify anthrax directly from blood samples, potentially bypassing the need for bacterial culture. This approach not only speeds up the diagnostic process but also improves disease monitoring and management. This is particularly important for regions like southern Africa, where anthrax poses a significant threat to wildlife health. Our work contributes to better conservation efforts and a deeper understanding of how to control anthrax outbreaks effectively.

## Introduction

Anthrax is an ancient zoonotic disease with a documented history dating back to biblical times [1]. While the disease affects many host species, herbivorous mammals are most susceptible, with fatalities often observed in wildlife and livestock. In addition, humans are susceptible to anthrax infections, and cases occur largely due to the handling or consumption of carcasses, infected meat, and hides [2,3]. Anthrax is generally known to be caused by *Bacillus anthracis*, which is an aerobic or facultative anaerobic, non-motile, Gram-positive, rod-shaped bacterium that produces endospores. This bacterium occurs in two forms, the spore form and the vegetative form [4]. The virulence factors of *B*. *anthracis* are encoded on two plasmids: pXO1, which is responsible for the production of the toxins, and pXO2, which synthesizes the poly-ɣ-D-glutamic acid capsule [5,6]. The pXO1 plasmid contains genes responsible for the production of protective antigen (PA, also referred to as BAPA; [3]), lethal factor (LF) and edema factor (EF) proteins. These proteins are grouped as A_2_B-toxins. The A components, which consist of the EF or LF, bears the enzymatic activity [7–9]. The B component consists of PA, which is the receptor-binding component of the lethal toxin (LT) and edema toxin (ET), and the courier of LF and EF respectively, into the host cells [8–11].

For a century, identifying anthrax and its causative agent, *B*. *anthracis*, relied on microbiological culture, microscopy, and biochemistry. Recently, new hypotheses about the disease’s presentation, prevention, and infective organisms have emerged in Africa [12–15]. There have been reports of serological cross-reactivity between pathogenic and non-pathogenic *Bacillus* spp. [16,17], in the high-incidence northern Kruger National Park (KNP). [18,19]. Anti-PA and LT-neutralizing antibodies were also detected at higher rates than expected in animals from southern KNP, a low-incidence area [19]. We hypothesized that animals might be reacting to “anthrax-like” microbes with genes similar to *B*. *anthracis* [19]. Additionally, the discovery of anthrax cases caused by *B*. *cereus* biovar *anthracis* (Bcbva) in West and Central Africa [20] prompted us to reassess the robustness of diagnostic tools currently used for anthrax surveillance in southern Africa. Furthermore, anthrax-like illnesses attributed to atypical strains of *B*. *cereus* and Bcbva. have been reported in animals, some of which include chimpanzees (*Pan troglodytes*), gorilla (*Gorilla gorilla*), elephants (*Loxodonta africana*), cattle (*Bos taurus*), and goats (*Capra hircus*) [12,15,20–24] in West and Central Africa. Norris *et al*.[25] also reported Bcbva in archival bones and teeth of monkeys from Côte d’Ivoire.

Different methods have been employed in the diagnosis of bacterial zoonoses such as *B*. *anthracis* over the years. These methods include the identification of bacterial culture isolates, microscopic examination of blood smears, molecular diagnosis targeting pathogen genetic markers and serological identification employing antibodies targeting antigens produced by the pathogen. The success of these techniques, however, depends largely on the specificity and sensitivity of the test being employed [26]. *Bacillus anthracis* is in the phylum Firmicutes, family Bacillaceae, and belongs to the group referred to as the *B*. *cereus* group. The *B*. *cereus* group consists of 11 *Bacillus* species (*B*. *anthracis*, *B*. *cereus*, *B*. *thuringiensis*, *B*. *mycoides*, *B*. *pseudomycoides*, *B*. *weihenstephanensis*, *B*. *cytotoxicus*, *B*. *toyonensis*, *B*. *gaemokensis*, *B*. *manliponensis* and *B*. *bingmayongensis*) that have closely related phylogenies [27–29] as reflected by high similarities in 16S rRNA gene sequences [24,30] and other genetic markers such as *gyrB* within the *B*. *cereus* group [31]. These species also differ in their aetiology, pathogenesis, clinical manifestations and host preferences [28,32–35]. The *gyrB* gene encodes the B subunit of DNA gyrase, an enzyme critical for DNA replication, transcription, and repair in bacteria. The *gyrB* gene sequence is highly conserved among bacterial species but varies enough to distinguish between them [31,36]. Studies have shown that sequencing of the gyrB gene can offer higher resolution than the more commonly used 16S rRNA gene in differentiating closely related bacterial species [37,38].

The initial step in the confirmation of *B*. *anthracis* in an anthrax-suspected carcass is the examination of blood smears stained with either Gram or Giemsa stain to view the rod-shaped bacterium [3]. The presence of encapsulated square-ended rod-shaped bacteria that react to the polychrome methylene blue stain indicates the presence of *B*. *anthracis* and warrants a sample to be sent to a reference laboratory for confirmation [3]. To confirm the presence of *B*. *anthracis* in the reference laboratories, the samples are cultured on blood agar to check for colony morphology [3], and the absence of haemolysis and sensitivity to penicillin and bacteriophages [39,40]. For additional verification, real-time/quantitative PCR (qPCR) is conducted for the presence of *pagA* with BAPA probe, *lef* [3], *capB* [3] and/or Ba-1 [41] genes that encode for virulence factors including the PA and capsule as well as *B*. *anthracis* chromosome, respectively. The qPCR targeting *pagA* with BAPA probe (pXO1) [3] and *capC* (pXO2) regions [3] used by Lekota *et al*.[42] has been reported to be inadequate for distinguishing closely related *Bacillus* species from anthrax outbreaks, while Zincke *et al*. [41] used *capB*, *lef* and Ba-1 targets to differentiate *B*. *anthracis* from *B*. *cereus sensu stricto*. Although the Ba-1 marker seems distinctive to *B*. *anthracis*, its validation has been limited to *B*. *anthracis* strains of *B*. *cereus sensu stricto*, similar to the case of *lef* [41,43]. Molecular targets typically focus on specific chromosomal regions unique to* B. anthracis* and virulence factors located on the pXO1 or pXO2 plasmids, which serve as virulent markers [3,42,44]. This approach arises from the high genomic similarity among closely related *Bacillus* spp. [45,46], as well as the presence of *B. anthracis* virulence plasmids or their components in other closely related species [15,47]. One of the most common diagnostic markers used in the detection of Bcbva is the genomic island IV (GI4) which is unique to Bcbva [41]. Over the last decades, there have been calls to move away from culture identification of *B*. *anthracis* in a bid to reduce biosafety risk and avoid proliferation [48]. Thus, the ultimate goal of this study was to investigate the best practices using culture-free methods for the diagnosis of anthrax.

In a typical *B. anthracis* investigation, the presence of *B. cereus* group species that are not *B. anthracis* is often regarded as contamination. Furthermore, the absence of genes associated with both the pXO1 and pXO2 plasmids further reinforces the perception that these closely related species are less significant and typically associated with less severe disease [49]. However, toxigenic *B*. *cereus* are known to have pXO1-like plasmids and other capsule-encoding plasmids that are not pXO2, and *B*. *cereus* can cause foodborne infections without either pXO1 or pXO2 plasmids [50]. As a result, microbes that lack the *B*. *anthracis*-specific chromosomal gene (Ba-1) or the pXO1 and pXO2 plasmids can be readily overlooked. In recent years, there have been reports of atypical *B*. *cereus* strains that are known to cause anthrax-like infections in both humans and animals [51] with very similar genes to those found on pXO1 and pXO2 plasmids found in *B*. *anthracis* [47].

Anthrax is endemic in KNP, and park personnel employ a passive surveillance system where blood smears are collected from any deceased animal and stored in an archival collection. We utilised blood smears from the collection, covering the years 2012–2020. This period encompassed known anthrax outbreaks from 2012 to 2015 [19,52]. From these outbreaks, *B*. *anthracis* bacilli were initially identified using the microscopic evaluation of blood smears from wildlife carcasses in KNP with follow-up collection of bone, hair, and tissue samples from positive carcass sites in previous study [52]. In this study, 25 *B*. *anthracis* isolates previously confirmed using microbiology and PCR from tissue samples linked to positive blood smears served as positive controls. Our investigation focused on employing microscopy, culture, and molecular markers, including real-time/quantitative polymerase chain reaction (qPCR), to identify *B*. *anthracis* and distinguish it from *B*. *cereus* or other closely related microbes. Specifically, we examined: 1) the performance of five molecular markers currently in use (*pagA* with BAPA probe, Ba-1, *lef*, *capB*, GI4) to identify *B*. *anthracis* from other bacteria using cultures of blood smears; (2) the performance of five molecular markers to identify *B*. *anthracis* from *B*. *cereus* and other closely related bacteria; and 3) we evaluated the agreement between anthrax diagnoses based on blood smear microscopy versus molecular techniques.

## Materials and methods

### Ethics statement

This study was reviewed and approved by University of Pretoria Research Ethics Committee, Animal Ethics Committee (REC 049–21), Department of Agriculture, Forestry and Fisheries (DAFF) in South Africa (Ref 12/11/1/1/6 (2382SR)) in South Africa, South African National Parks (SANParks), South Africa (Ref: SS318).

### Study area

**Fig 1:**
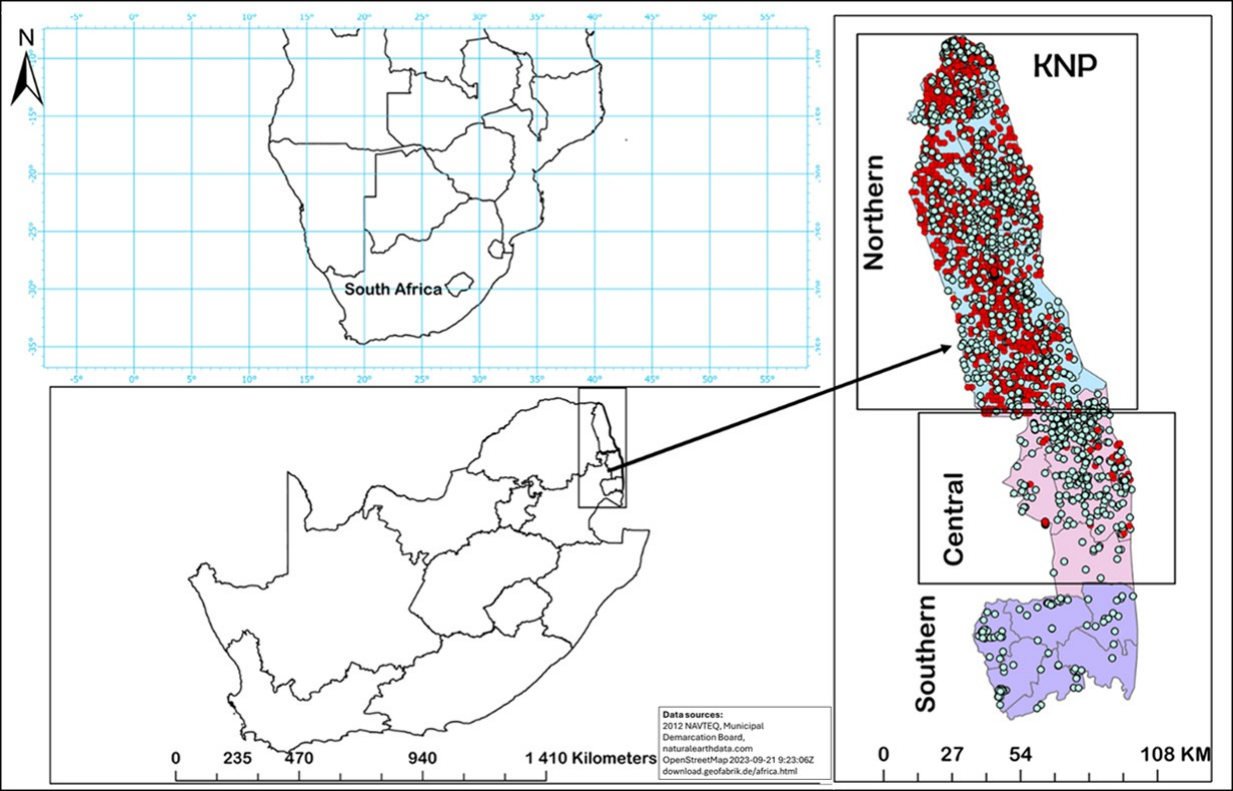
The study area, Kruger National Park (KNP), is located in South Africa. The map of 210 KNP divides the park into three regions, with the distribution of 1708 animal mortalities (S1 Table) investigated in this study are shown as dots; presumptive anthrax positive cases, identified through microscopic examination of blood smears, are marked with red dots, while green dots indicate anthrax-negative mortalities. South Africa provincial and municipal map obtained from africa-latest.osm.pbf. KNP shape files were obtained from from Navteq (2024). The Africa map was obtained from the natural earth data (https://www.naturalearthdata.com/downloads/10m-cultural-vectors/10m-admin-1-states-provinces/)).

The KNP (19,485 km^2^; Fig 1) is situated in the northeastern part of South Africa, bordering Mozambique and Zimbabwe. The northern half of KNP (Fig 1) is considered the anthrax endemic region, where most of the anthrax mortalities have been reported [19,53]; this region is classified as semi-arid and is highly wooded with some grassland savannah [54]. KNP has variable elevations, with Pafuri (found in the northernmost part of KNP; 22.4206° S, 31.2296° E) having lower elevation floodplains and mountains towards the northwestern part of the park. In KNP, the high anthrax incidence (endemic) area extends from Pafuri to Shingwedzi (23.1167° S, 31.4333° E) in the north, and the low incidence area extends from Skukuza (24.9948° S, 31.5969° E) to Crocodile Bridge (25.3584° S, 31.8935° E) in the south (Fig 1).

### Sample preparation and DNA extraction

Archival blood smears can be an important resource for retrospective studies and for retrieving pathogens like *B*. *anthracis* that can remain viable for years [52,55]. In KNP, as part of the passive surveillance by the Skukuza State Veterinary Services, blood smears have been collected from all carcasses discovered during field surveys. Two smears were collected per carcass, one of which is stained (with Giemsa), while the other remains unstained. Metadata captured at the carcass sites include the date, Global Positioning System (GPS) coordinates, locality, species, and sex. These smears were first examined at the time of collection and then stored at room temperature since collection. Aminu, Lembo [56] demonstrated that Azure B staining is more robust, consistent and has a higher sensitivity compared to Giemsa only, without Azure B and Polychrome Methylene Blue (PMB) stains. The Giemsa stain used in this study contained Azure B.

A total of 1708 Giemsa-stained blood smear slides (from wildlife mortalities recorded 2012–2020; S1 Table) were examined by microscopy at 1000X magnification for the presence of square-ended cells indicative of *B*. *anthracis*. All phenotypic confirmation of *B*. *anthracis* by microscopy and plate assays were performed as described by the World Health Organization [3]. Each slide was examined by two examiners. The selection of smears from this time period (2012–2020) was based on the findings of Hassim [52] who demonstrated that isolate recovery reduced with age of the smears. We used the selected corresponding unstained smears (N = 1708) for additional genetic and microbiological work.

The unstained blood smears from each mortality were scraped into a collection plate and transferred into a 1.5 mL centrifuge tube using a sterile scalpel. The smear scrapings were added to 200 μL of phosphate buffered saline (PBS; Thermo Scientific, MA, USA) and divided into two aliquots. The first aliquot was subjected to automated DNA extraction (QIAcube, QIAGEN GmbH, Hilden, Germany) using the DNA Blood Mini kit (QIAGEN QIAmp, QIAGEN GmbH, Hilden, Germany) and the manufacturer’s instructions for DNA extraction from blood were followed. The second aliquot was inoculated on 5% sheep blood agar (SBA) and incubated overnight at 37°C for use in the morphological identification of bacterial colonies, as described by Parry, Turnbull [57]. On each plate, all bacterial colonies demonstrating different colony morphology were selected and treated as different isolates. All isolates identified were further sub-cultured onto 5% SBA to obtain pure cultures and check for the presence of haemolysis and colony morphology. The purified isolates were further subjected to gamma-phage and penicillin sensitivity tests. Isolates that did not present with a *B*. *anthracis* characteristic phenotype were retained and screened using molecular methods. DNA extraction from pure isolates was performed using the Pure link Genomic DNA kit (Thermo Fisher Scientific, MA, USA) as prescribed by the manufacturer.

If a mortality was identified as positive for *B*. *anthracis* based on microscopy, a follow-up sample (soil, bone and/or tissue) from the carcass site was collected as soon as possible (if GPS coordinates were available). From these additional samples, 25 isolates confirmed to be *B*. *anthracis* based on PCR, morphology, microscopy, lack of haemolytic activity, gamma-phage and penicillin sensitivity were used in this study as internal positive controls. These controls serve as a benchmark to verify that the assays are functioning correctly and to validate the results obtained from the experimental samples. Additionally, these controls were obtained at or close to the carcass site and from other tissues or samples of the respective animals, providing a more accurate reference for comparison.

### Microscopic examination of bacterial isolates derived from blood smears

For the blood smear scrapings that yielded bacterial growth, the colonies were subcultured (to obtain pure colonies) and transferred directly to a microscope slide, and 5 μL of saline was added, emulsified, and spread evenly on the slide. The slide was allowed to dry and fixed with 95% methanol (Merck KGaA, Darmstadt, Germany) for one minute. The methanol was allowed to dry and a Gram stain was conducted to visualise the presence of Gram positive rods. The presence of Gram positive, square-ended rods, typical of *B. anthraci*s at 1000X magnification as described above. Subsequently, to determine encapsulation, polychrome methylene blue stain was performed.

### Quantitative polymerase chain reaction (qPCR) on bacterial isolates derived from smears

The qPCR was performed on two different sample sets. First, cultured isolates from one of the two aliquots of the blood smears (506 isolates from 113 smears) were screened, targeting 5 genetic markers for *pagA* with BAPA probe, Ba-1, *lef*, GI4 and *capB* in a stepwise manner. All isolates were screened even if they were not phenotypically *B*. *anthracis*. The isolates were first screened with the SYBR Green PCR assays using primers and targets in Table 1 as described by W.H.O. [3] and the manufacturer (CelGREEN, Celtic Molecular Diagnostics, Cape Town, South Africa). Isolates that were positive with SYBR Green (n = 125) PCR assays on all the markers were then further confirmed using the TaqMan assay for targeting the Ba-1, *lef*, *capB*, GI4 targets (Table 1) as described by Zincke *et al*. [41] and the fluorescence resonance energy transfer (FRET) qPCR for BAPA (Table 1) [3]. The inclusion of the chromosomal marker, Ba-1 and GI4 in the assay was based on the premise that it enhances the specificity of the assay, as detailed by [58]. The reaction mixtures for the SYBR Green PCR assay, targeting the *pagA* with BAPA probe, Ba-1, *lef*, GI4, and *capB* primer sets, consisted of 0.5 μM of each primer. For Ba-1, *lef*, GI4, and *capB*, the mix included 1x SYBR Green (CelGREEN, Celtic Molecular Diagnostics, Cape Town, South Africa), while the *pagA* assay utilized FastStart Essential Green Master (Roche, Basel, Switzerland). Each mixture also contained 2 ng of DNA, resulting in a total volume of 20 μL per reaction. Cycling conditions were: a pre-incubation at 95°C for 10 min (20°C/sec ramp), followed by 45 cycles of 95°C for 10 sec and 55°C for 20 sec (both at 20°C/sec ramp), then 72°C for 30 sec (20°C/sec ramp) with signal capture post-annealing. Denaturation involved an immediate 95°C step, cooling to 40°C for 30 sec (both at 20°C/sec ramp), then 80°C instantly with a 0.1°C/sec ramp for continuous signal reading. The process concluded with a cool-down to 40°C for 30 sec (20°C/sec ramp). The assay was performed using the QuantStudio 5 Real-time PCR system (Thermo Fisher Scientific, MA, USA). Isolates that were positive for *pagA* (n = 14) were selected for further identification using gyrase B (*gyrB*) gene PCR as described by [31]. We selected these isolates that were *pagA* PCR positives as it has been hypothesised that closely related bacterial species might be responsible for the anti-PA serological reaction observed in anthrax nonendemic regions [19]. The cycle threshold (CT) cutoff was established at 35 for Ba-1 and GI4, as well as for *pagA*, *lef*, and *capB* [41,42,52]. For *B*. *anthracis*, we used *B*. *anthracis* Vollum strain as the positive control, and the 25 smear samples confirmed to be *B*. *anthracis* in the study of Hassim [52] were used as internal controls. We obtained a positive control (DNA from pure culture) for Bcbva from the Robert Kock Institute, Germany.

**Table 1:** Primers, probes and gene targets for the detection of *Bacillus anthracis* from bacterial isolates cultured from blood smear samples from wildlife mortalities in Kruger National Park, South Africa by quantitative polymerase chain reaction (qPCR) assays. *Bacillus anthracis* protective antigen (BAPA), lethal factor (*lef*), chromosomal marker (Ba-1 and Genomic island 4: GI4) and the capsule region (*capB*) were used as molecular markers in this study. FRET stands for fluorescence resonance energy transfer.

Primer/Probe (5′–3′)	Chemistry	Target	Reference
Forward ‐ GTACATCTTCTAGCTGTTGCAA Reverse–ACGTAGGAAGACCGTTGATTA Probe ‐ VIC-CGTTGTTGTGTATTTG-MGB	TaqMan	Ba-1	[41]
Forward–TAAGCCTGCGTTCTTCGTAAATG Reverse–GTTCCCAAATACGTAATGTTGATGAG Probe ‐ NED-TTGCAGCGAATGAT-MGB	TaqMan	*capB*	
Forward–CACTATCAACACTGGAGCGATTCT Reverse–AATTATGTCATCTTTCTTTGGCTCAA Probe ‐ Cy5-AGCTGCAGATTCC-MGB	TaqMan	*lef*	
Forward ‐ GGAGATATTAACAAGAGATGGATTGGA Reverse ‐ CAGTAGGCTTGTCTGCTCTAATAAAATT Probe ‐ FAM-ACATGCCAGCGTTTTTTGCCTCTACACA-BHQ1	Taqman	GI4	
Forward–CGGATCAAGTATATGGGAATATAGCAA Reverse ‐ CCGGTTT AGTCGTTT CTAATGGAT BAPA-FL ‐ TGCGGTAACACTT CACTCCAGTTCGA-X BAPA-LCRed 640 - CCTGTATCCACCCTCACTCTT CCATTTT C-P	FRET	*pagA* with BAPA probe	[3]

### qPCR from direct scrapings of blood smears

Secondly, the 1708 DNA samples obtained from blood smear scrapings were screened for the presence of the pXO1 plasmid, with qPCR assays targeting the *pagA* and *lef*, as well as pXO2 plasmid targeting *capB*. We also screened for the chromosomal markers Ba-1 of *B*. *anthracis* and GI4 region for Bcbva. To determine the presence of *pagA* with BAPA probe, qPCR was conducted using the FRET on the Light Cycler Nano (Roche, Basel, Switzerland). For the TaqMan PCR assay, the reaction conditions were standardized to a 20-μL mixture containing 1 μL of the DNA template, 1x concentration of PrimeTime Gene Expression Master Mix (IDT, Coralville, IA, USA, Cat No. 1055772), along with primers and probes as listed in Table 1.

The thermal cycling conditions for TaqMan PCR assays were set as follows: an initial denaturation at 95°C for 3 minutes, followed by 45 cycles of denaturation at 95°C for 20 sec and annealing/extension at 60°C for 30 sec. For *lef*, Ba-1, *capB* and GI4, qPCR TaqMan assay was performed using the QuantStudio 5 Real-time PCR system (Thermo Fisher Scientific, MA, USA). Two duplex assays were created for the simultaneous detection of FAM- and VIC-labeled probes. The first duplex targeted Ba-1 and GI4 markers for species identification, while the second targeted *lef* and *capB* virulence markers from pXO1 and pXO2 plasmids, respectively. To prevent spectral overlap in the QuantStudio 5 instrument, colour compensation was conducted with FAM and VIC probes, applying the results to duplex assay data. Tests included all 25 confirmed positive *B*. *anthracis* strains (∼1 ng DNA), and specificity checks involved DNA from Bcbva, and *B*. *cereus* ATCC 3999. The C_T_ cutoff for positive samples was set at 35 for all the markers [42,52].

### Molecular identification and phylogenetic analysis on bacterial isolates from smears

The 14 bacterial isolates from blood smear scrapings that tested positive for *pagA* with BAPA probe by the two qPCR approaches were subjected to additional molecular and phylogenetic analysis. The gyrase B (*gyrB*) and *pagA *PCR products were sequenced for molecular taxonomic identification of the isolates. The PCR fragments of the *gyrB* gene of the selected isolates (n = 14), including 4 *B*. *anthracis* isolates based on microbiology (square-ended bacilli, colony morphology, penicillin and gamma phage sensitive), were sequenced at Inqaba Biotechnical Industries (Pty) Ltd., Pretoria, South Africa. A BLAST search query was performed to compare the *gyrB* nucleotide sequences from the *Bacillus* isolates with publicly available GenBank sequences in NCBI (http://www.ncbi.nlm.nih.gov; accessed on 08, March, 2023). Multiple sequence alignments of the mined *gyrB* reference sequences and *Bacillus* spp. strains sequenced in this study were performed using BioEdit 7 [59] and using the algorithm found in Clustal W MEGA11 as described by Tamura, Stecher [60]. With this alignment, we inferred the phylogenetic relationships of the *Bacillus* spp. isolates with respect to other related species and *B*. *anthracis*. The p-distance model was used to generate a neighbour-joining tree with 1000 bootstrapped replicates, using the MEGA 11.0 software [60], and the phylogenetic tree was visualised using ITOL 5.0 [61].

The 240 bp amplicons, targeting the *pagA* gene and detected using the BAPA probe [3], were analysed on the presumptive *Bacillus* spp. isolates bearing the following sample numbers: AX2015 (1122; 1136; 1152; 1511 and 1277A) and AX2016 (1708NH and 1800) and sequenced at Inqaba Biotechnical Industries (Pty) Ltd., Pretoria, South Africa. The BLASTn homology searches of the sequences were performed to assess homologous hits against the *pagA* region of the *B*. *anthracis* GenBank sequences available in NCBI [62]. Multiple sequence alignments of the *pagA* (BAPA) probe region were performed using BioEdit 7 [59]. The isolates and/or PCR fragments that failed quality control (low-base calling during sequencing: the sequences where at least 90% of the nucleotides achieved a Phred score of less than 30) were excluded from this analysis.

The analysis of the *gyrB* gene was performed to provide a broader phylogenetic context for the bacterial isolates identified as *Bacillus anthracis* based on *pagA* sequencing and BAPA probes. The BLASTn homology searches of the *pagA* region were used to assess homologous hits against *B*. *anthracis* sequences available in GenBank (NCBI, 2023). This dual approach—first targeting *pagA* for *B*. *anthracis* identification and then conducting broader *gyrB* phylogenetic analysis—was necessary to confirm both species-level identification and the phylogenetic relationships within these closely related species.

### Data analysis

#### Performance analysis of markers on bacterial isolates

All results for the qPCR of the isolates were presented as counts and percentages. To assess the performance of these molecular markers, we analysed 80 isolates that tested qPCR positive for individual markers or combinations of molecular markers using the probe-based approach. We used culture, microscopy, penicillin sensitivity, and gamma-phage sensitivity results as the gold standard (true positive/negative) for comparison with the assays [3]. For the isolates that tested positive for any of the markers, we calculated the specificity, which detects true negative, and the sensitivity, which detects true positive [63]. We also calculated the positive predictive value (PPV); probability that *B*. *anthracis* is present when the test is positive, the negative predictive value (NPV), probability that *B*. *anthracis* is absent when the test is negative, and the accuracy, which refers to the overall probability that a case is correctly classified [63]. Results for specificity, sensitivity, and accuracy were presented in percentages and confidence intervals (CI) which are Clopper-Pearson CI [64] and the CI for the predictive values was calculated using the log method as described by Altman, Machin [65].

#### Analysis of smears and direct qPCR of scrapings

The outcomes of the qPCR and microscopic examination of blood smears were represented as counts and percentages of positive samples. We evaluated the extent of agreement between the binary outcomes of the molecular tests and the results of the microscopic examination of the blood smears. This was done using a Cohen’s kappa (k) test [66]. For this analysis, kappa ≠ 0 implies that the extent of agreement between the two tests mentioned was significantly different from chance agreement. The measure of agreement was evaluated based on the criteria of Landis and Koch [67], where <0 = poor; 0.01–0.20 = slight; 0.21–0.40 = fair; 0.41–0.60 = moderate; 0.61–0.80 = substantial; 0.81–1.00 = almost perfect. Statistical analyses were conducted using R version 4.1.2 [68], and significance was evaluated with a threshold of alpha < 0.05.

## Results

### Isolation and identification of cultured samples

**Fig 2:**
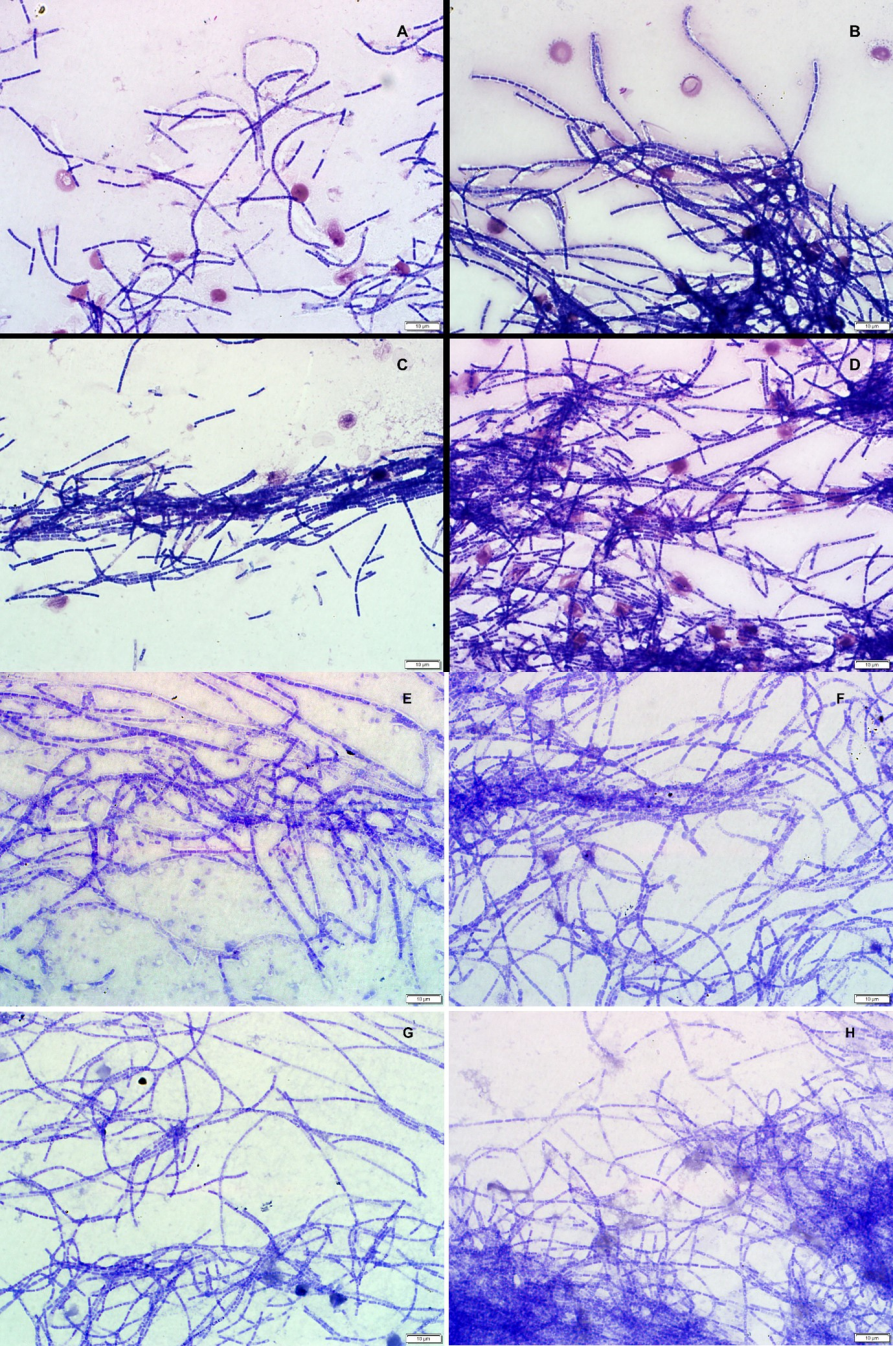
Microscopic examination of Gram and polychrome methylene blue-stained cultures from bacterial isolates collected from wildlife blood smears in Kruger National Park, South Africa, identified *Bacillus anthracis* based on morphology. Images show square-ended bacilli: (A) Isolate AX2015-1270, (B) AX2015-1277A, (C) AX2015-1152, and (D) AX2015-1136. Encapsulation is visible in (E) AX2015-1270, (F) AX2015-1277A, and (G) AX2015-1152, except in (H) AX2015-1136, which lacked a capsule.

Out of the 1708 blood smear scrapings that were cultured, only 113 samples had bacterial growth from which a total of 506 pure colonies were isolated (some smears yielded multiple different bacterial colony forming units). Only 4/506 colonies demonstrated morphological features that were consistent with those of *B*. *anthracis* (AX2015-1270, AX2015-1277A, AX2015-1152, and AX2015-1136). The colony morphology and structure of the four isolates on 5% SBA demonstrated non-heamolytic features, forming typical white-gray colonies with an oval, slightly granular appearance. The Gram-stained isolate smears from the 4/506 positive samples showed square-ended bacilli that are classical to *B*. *anthracis* (Fig 2). Upon examination of the polychrome methylene blue stained smears, the identified *B*. *anthracis* isolates appeared square-ended and encapsulated (with the exception of AX2015-1136, Fig 2H). The remaining 502 isolates from this study failed on all or some of the criteria (colony morphology, granularity or colour, hemolysis, capsule detection, penicillin and gammaphage sensitivity). The smear samples that failed to produce any colonies (including 25 positive internal controls) were established, suggesting the *B*. *anthracis* endospores were no longer viable to germinate on culture media.

### Molecular analyses of bacterial isolates

**Fig 3:**
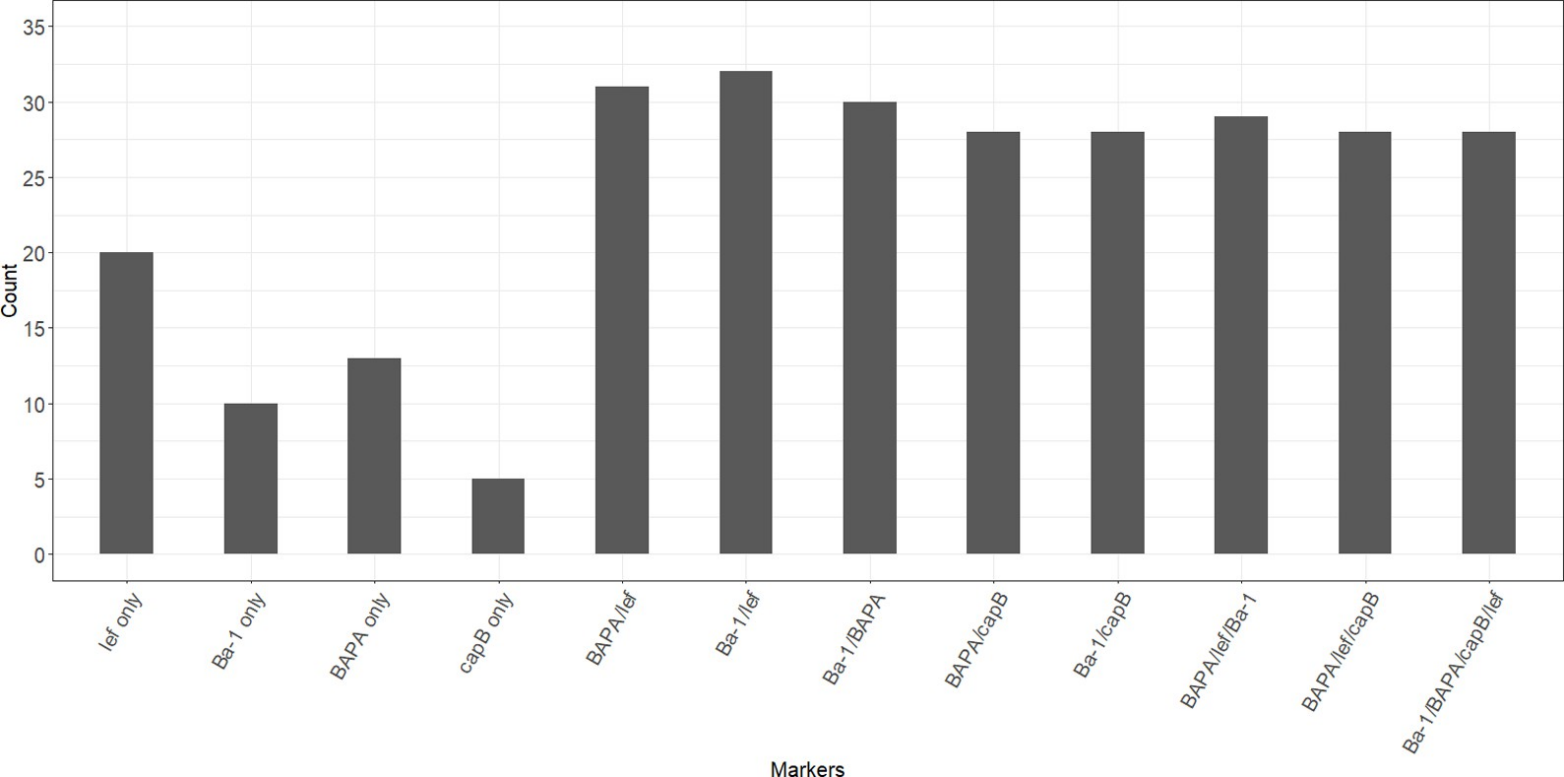
Bar plots displaying the counts of bacterial isolates from blood smears testing positive for *Bacillus anthracis* using various molecular markers or combinations. Results are based on qPCR of 80 positive isolates, collected from wildlife mortalities in Kruger National Park, South Africa, between 2012 and 2020. Molecular markers include *Bacillus anthracis* protective antigen (*pagA* with BAPA probe), lethal factor (*lef*), chromosomal marker (Ba-1), and capsule region (*capB*).

Of the 506 bacterial isolates, only 125 (24.7%) tested positive for one or more of the molecular markers (Ba-1, *lef*, *pagA* with BAPA probe, *capB*) using SYBR Green. The probe-based FRET and Taqman qPCR assays detected 80 of the 125 isolates were positive (Fig 3). The use of the "+" symbol in our context signifies the strategic combination of markers. The combination of BAPA + *lef* + Ba-1 successfully identified the four *B*. *anthracis* isolates that were confirmed through culture and microscopy. This combination yielded exclusively these four positives representing 5% (n = 4) of the total.

### Microbiological screening of the 14 bacterial isolates that were confirmed to be positive on *pagA* (with BAPA probe)

The most commonly used genetic marker for the diagnosis of *B*. *anthracis* is *pa*gA with BAPA probe, and the use of this marker is recommended by the W.H.O. [3]; however, in our samples this marker was not specific to *B*. *anthracis*. Following a microbiologic screening of the 14 samples that tested positive for *pagA* by qPCR (S1 Fig), 7/14 showed penicillin sensitivity, while only the samples that were identified as *B*. *anthracis* (i.e., by colony morphology, capsule staining, heamolysis and molecular markers) showed gamma phage sensitivity (Table 2). Only 2 of the 14 samples were haemolytic, and the *B*. *anthracis* strains were all non-haemolytic (Table 2). Three of the four *B*. *anthracis* strains tested positive for all four markers (*pagA* with BAPA probe, Ba-1, *lef* and *capB*), while the one *B*. *anthracis* only demonstrated detection of the Ba-1, *pagA* with BAPA probe and *lef* markers. This is consistent with the microscopic analysis indicating the absence of a capsule. Most marker combinations of chromosome and toxin genes, as well as combinations of different toxin gene targets, misclassified *B*. *anthracis*. A combination of microscopy and molecular qPCR chromosome and toxin targets accurately detected *B*. *anthracis*. In contrast, using a capsule target underestimated *B*. *anthracis* due to the anomalous loss of the capsule encoding pXO2 (Fig 3).

**Table 2:** Results of penicillin and Gamma phage sensitivity, morphology and quantitative polymerase chain reaction (qPCR) for the Taqman and FRET probe-based chemistry using four different molecular markers (*Bacillus anthracis* protective antigen (BAPA refer to *pag*A region with BAPA probe/sequence), lethal factor (*lef*), and capsule (*capB*) genes and the chromosomal region (Ba-1)) for selected bacterial isolates collected from the scrapings of carcass blood smears from 2012–2015 from Kruger National Park, South Africa. These 14 isolates were those that tested positive for the *pagA* region with BAPA sequence. Genus identity was based on the *gyrase B* sequence data. The probe-based approach was only conducted on isolates that were positive with SYBR Green assay.

Markers		Ba-1		*lef*		BAPA		*capB*		Sensitivity and morphology		
Sample ID	Genus	SYBR Green	Probe	SYBR Green	Probe	SYBR Green	Probe	SYBR Green	Probe	Penicillin	Gammaphage	Haemolysis
AX2015-1122	*Peribacillus*	+	-	-	+	+	+	-	-	+	-	-
AX2016-1800	*Peribacillus*	+	+	-	+	+	+	-	-	+	-	-
AX2015-1277A^*^	*Bacillus*	+	+	+	+	+	+	+	+	+	+	-
AX2015-1136^*^	*Bacillus*	+	+	+	+	+	+	-	-	+	+	-
AX2015-1152^*^	*Bacillus*	+	+	+	+	+	+	+	+	+	+	-
AX2015-1270^*^	*Bacillus*	+	+	+	+	+	+	+	+	+	+	-
AX2016-1771A	*Bacillus*	-	-	+	+	+	+	-	-	-	-	+
AX2014-1037	*Bacillus*	-	-	+	+	+	+	-	-	-	-	+
AX2016-1708NH1	*Priestia*	+	+	-	-	+	+	-	-	+	-	-
AX2015-1511Nm	*Priestia*	+	+	-	+	+	+	-	-	-	-	-
AX2015-1511BE	*Priestia*	+	-	-	-	+	+	-	-	-	-	-
AX2016-1705	*Priestia*	-	-	-	-	+	+	-	-	-	-	-
AX2013-415	*Priestia*	+	-	+	-	+	+	-	-	-	-	-
AX2014-721	*Priestia*	-	-	+	+	+	+	-	-	-	-	-

Asterisk (*) on sample IDs denotes *Bacillus anthracis*. The *Priestia* and *Peribacillus* species were previously identified as *Bacillus*: however, Bergey’s manual [69] for the nomenclature of bacteria still refers to these as *Bacillus*.

### Performance of the pXO1, pXO2 gene and chromosomal markers

The different molecular markers alone and in combination demonstrated varying specificity, sensitivity, PPV, NPV, and accuracy (Table 3). The 80 isolates identified as positive for BAPA by the qPCR/probe approach in this study include the 4 *B*. *anthracis* isolates identified from the smears. The *lef* marker demonstrated the lowest specificity and accuracy (51.2% and 72.5%, respectively; Table 3). Specificity and accuracy for Ba-1, *pagA* with BAPA probe, and *capB*, for qPCR were all above 60.0%, with Ba-1 having the lowest and capB having the highest specificity and accuracy (Table 3). The combination of markers increased the specificity and accuracy of these markers. Combinations of Ba-1+*lef*, BAPA+*lef*, and Ba-1+BAPA showed specificities and accuracies of over 95% (Table 3). The specificity and accuracy were 100% and 98.8%, respectively, for all combinations of Ba-1+*capB*, BAPA+*capB*, Ba-1+BAPA+*capB*+*lef*, and BAPA+*lef*+*capB*, however, with a sensitivity of 96.55% (Table 3). The combination of BAPA+*lef*+Ba-1 showed a specificity, sensitivity, and accuracy of 100% which is the overall probability that a case is correctly classified.

**Table 3:** Performance of quantitative polymerase chain reaction (qPCR) probe-based diagnostic assays for detecting *Bacillus anthracis*, using individual markers as well as combinations of markers as assessed by their specificity, sensitivity, positive predictive value, negative predictive value and accuracy. All results are shown in percentages with confidence intervals (CI; 95%) in parentheses. The gold standard assessment of a true positive used in this analysis was culture identification, microscopy, and penicillin and Gamma phage sensitivity. Samples used here (n = 80) include *Bacillus anthracis* (Ba) and other bacterial species isolated from cultured blood smears obtained from wildlife mortalities in Kruger National Park, South Africa. *Bacillus anthracis* protective antigen (*pagA* region with BAPA probe), lethal factor (*lef*), chromosomal marker (Ba-1) and the capsule region (*capB*) were used as molecular markers in this study.

Markers	Specificity (CI) %	Sensitivity (CI) %	Positive predictive value(CI) %	Negative predictive value (CI) %	Accuracy (CI) %
Ba-1 only	75.61 (59.70–87.64)	72.50 (56.11–85.40)	74.36 (62.46–83.49)	73.81 (62.33–82.76)	74.07 (63.14–83.18)
*lef* only	51.22 (35.13–67.12)	72.50 (56.11–85.40)	59.18 (50.75–67.11)	65.62 (51.54–77.41)	75.00 (64.06–84.01)
BAPA only	67.50 (50.87–81.43)	72.50 (56.11–85.40)	69.05 (57.85–78.38)	71.05 (58.68–80.93)	70.00 (58.72–79.74)
*capB* only	90.00 (78.19–96.67)	96.67 (82.78–99.92)	85.29 (71.58–93.03)	97.83 (86.73–99.68)	92.50 (84.39–97.20)
Ba-1 + *lef*	94.12 (83.76–98.77)	100.00 (88.06–100.00)	90.62 (76.33–96.66)	100.00 (92.60–100.00)	96.25 (89.43–99.22)
BAPA + *lef*	96.08 (86.54–99.52)	100.00 (88.06–100.00)	93.55 (78.85–98.26)	100.00 (92.75–100.00)	97.50 (91.26–99.70)
Ba-1 + *capB*	100 (93.02–100.00)	96.55 (82.24–99.91)	100.00 (87.66–100.00)	98.08 (88.14–99.72)	98.75 (93.23–99.97)
BAPA + *capB*	100 (93.02–100.00)	96.55 (82.24–99.91)	100.00 (87.66–100.00)	98.08 (88.14–99.72)	98.75 (93.23–99.97)
Ba-1 + BAPA + *capB* + *lef*	100 (93.02–100.00)	96.55 (82.24–99.91)	100.00 (87.66–100.00)	98.08 (88.14–99.72)	98.75 (93.23–99.97)
BAPA + *lef* + *capB*	100 (93.02–100.00)	96.55 (82.24–99.91)	100.00 (87.66–100.00)	98.08 (88.14–99.72)	98.75 (93.23–99.97)
BAPA + *lef* + Ba-1	100.00 (93.02–100.00)	100.00 (88.06–100.00)	100.00 (88.06–100.00)	100.00 (93.02–100.00)	100.00 (95.49–100.00)
Ba-1 + BAPA	98.04 (89.55–99.95)	100.00 (88.06–100.00)	96.67 (80.64–99.51)	100.00 (92.89–100.00)	98.75 (93.23–99.97)

### *Bacillus* spp. differentiation using *gyrB*

**Fig 4:**
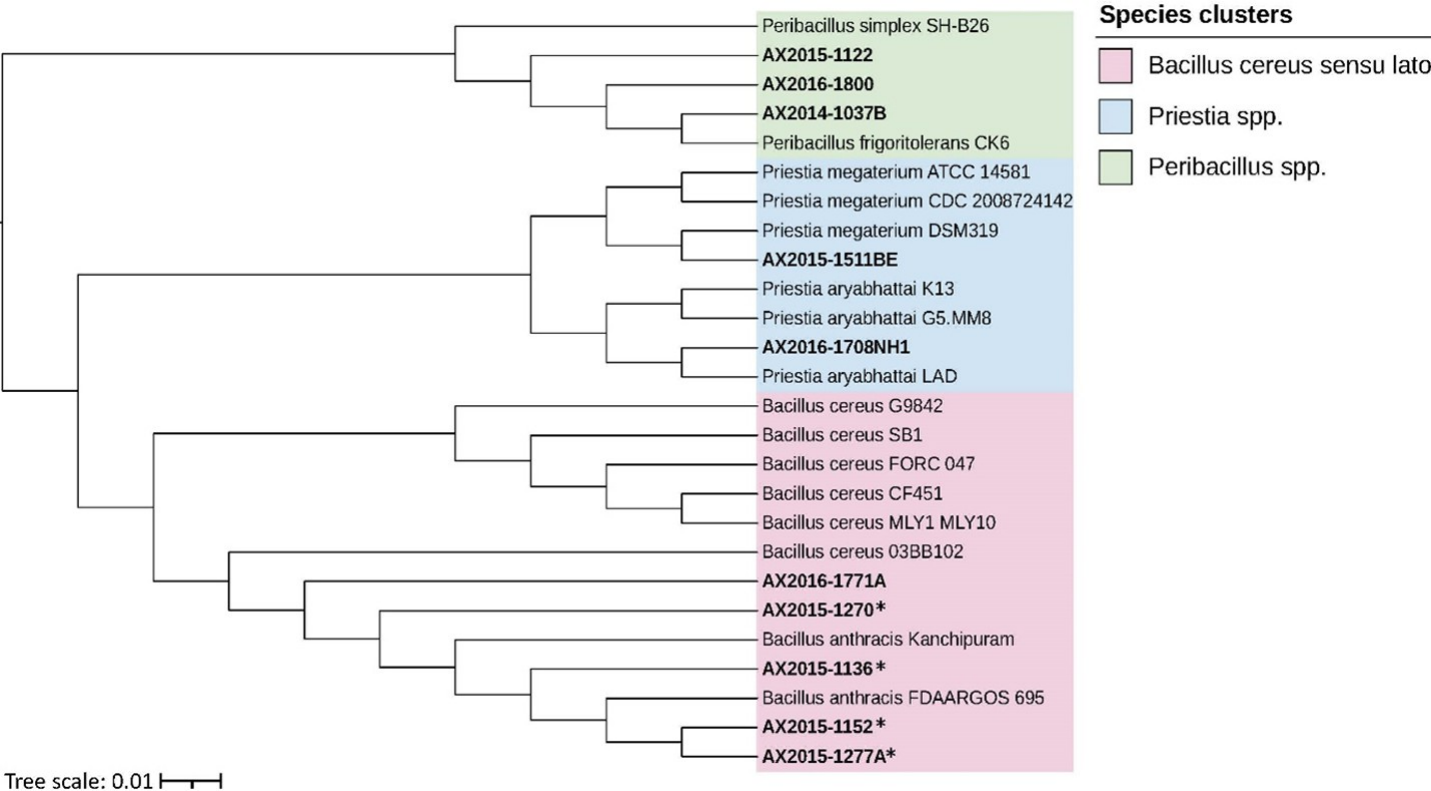
Phylogenetic tree of bacterial isolates from Kruger National Park, South Africa, based on the *gyrB* gene, constructed using the neighbor-joining method and p-distance model among three closely related genera (formerly *Bacillus* spp.). Isolates labeled with AX are from this study and were compared to the closest reference isolates from the National Center for Biotechnology Information (NCBI) (via BLASTn searches) of *Bacillus cereus sensu lato*, *Priestia* spp., and *Peribacillus* spp. The scale bar represents 0.010 substitutions per nucleotide position. Isolates confirmed as *B*. *anthracis* through microscopy, culture, molecular diagnosis, and sensitivity to penicillin and gamma phage are marked with an asterisk (*).

The BLASTn identification of the *gyrB* gene from the 14 selected bacterial isolates (i.e., those positive for *pagA* with BAPA probes/sequence) and subsequent phylogenetic analyses identified three genetic clusters, *B*. *cereus sensu lato* (comprising of *B*. *cereus* and *B*. *anthracis* found in this study), *Peribacillus* spp. and *Priestia* spp. (Fig 4). The latter two clusters were previously part of *Bacillus* and are recently proposed new genera [70] but are still documented as *Bacillus* spp. according to the Bergey’s’s manual [69]. The AX2015 strains (1152, 1277A, 1270 and 1136) grouped in the *B*. *cereus sensu lato* cluster with reference isolates *B*. *anthracis* (FDAARGOS 695 and Kanchipuram) as the closest related strains. The isolated AX2016-1771A strain clustered with *B*. *anthracis*, and also within a cluster including atypical *B*. *cereus*, although it had phenotypic characteristics with *B*. *cereus* as it was classified as haemolytic. AX2014-1037B; AX2015-1122 and AX2016-1800 grouped in the *Peribacillus* cluster (Fig 4). AX2015-1511BE grouped with *Priestia megaterium* reference strains, and AX2016-1708NH1 grouped closely with the *Priestia aryabhattai* reference strains (Fig 4). The following isolates AX2013-415, AX2014-721, AX2015-1511 Nm and AX2016-1705 were excluded from the tree as they failed to pass the quality control.

### The *pagA* (with BAPA probe) sequence alignment of the selected isolates

**Fig 5:**
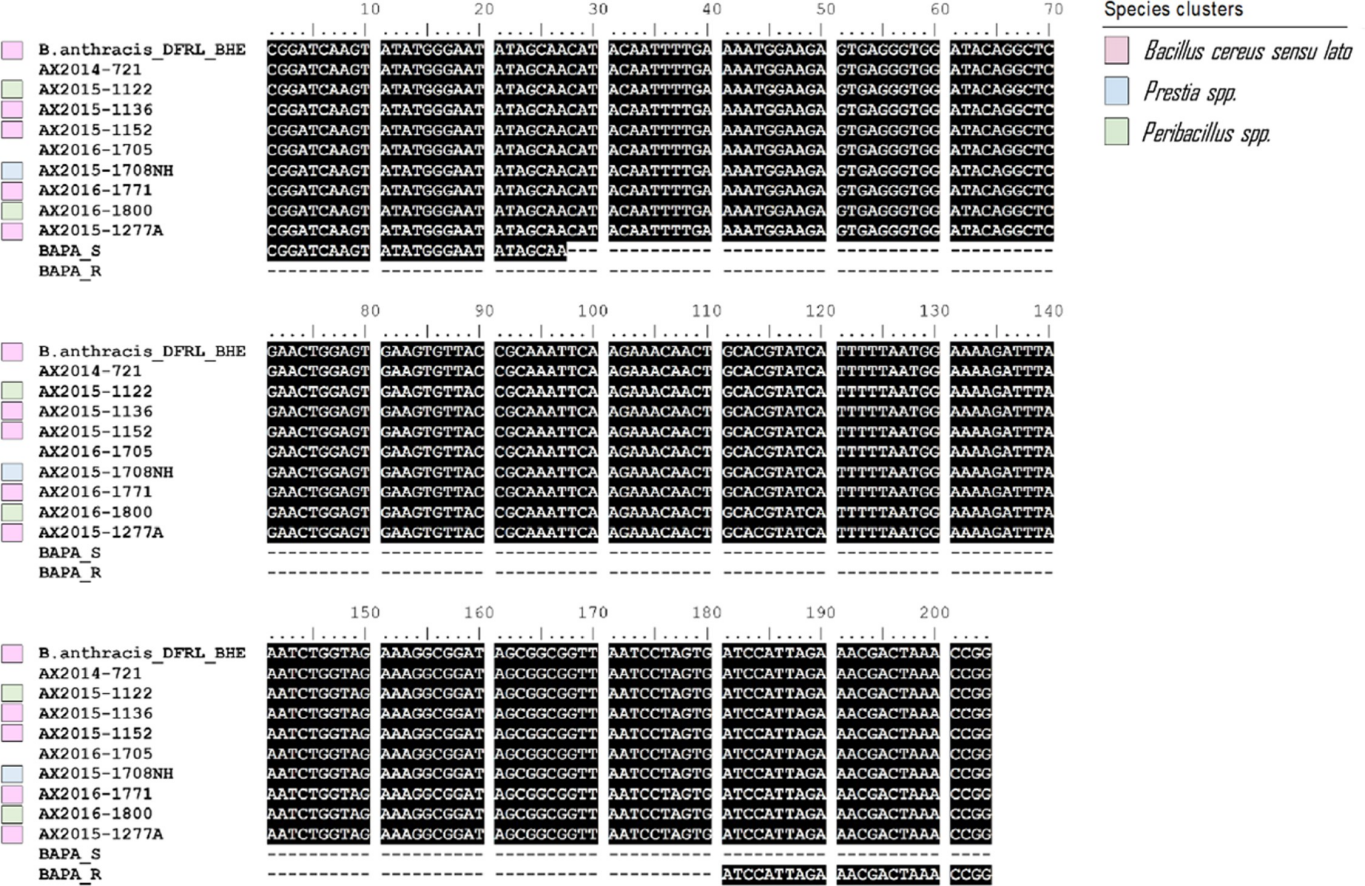
Multiple nucleotide sequence alignment of the *Bacillus anthracis* protective antigen (BAPA) region (targeting the *pagA* gene of *B*. *anthracis* with the BAPA probe) from isolates in this study (starting with AX) compared to the NCBI reference strain *B*. *anthracis* DFRL BHE-12. BAPA_S (Forward) and BAPA_R (Reverse) indicate the BAPA probe targeting sequences. Coloured blocks represent related species clusters, as shown in Fig 4. Sequences were aligned using BioEdit. Two isolates (AX2014-721 and AX2016-1705) lack colour blocks since their species clusters were not included in the previous analysis (Fig 4).

The 240 bp *pag*A (including BAPA probe) region of AX2014-721, AX2015 (1122; 1136; 1152;1277A) and AX2016 (1771A; 1705; 1800; 1708NH1) were aligned against the NCBI reference strain of *B*. *anthracis* DFRL BHE-12 *pagA* gene region and the BAPA probes (See Fig 5 with probe sequence) to confirm specific *pagA* binding. The results showed no difference in comparison to the reference *B*. *anthracis* strains and showed the *pagA* region was completely conserved across the isolates (Fig 5).

### Probe-based qPCR and microscopy results of scraped blood smears

DNA extracted from blood smears revealed that a substantial number of samples tested positive for at least one molecular marker (S1 Table). Among these, specific subsets tested positive exclusively for certain markers: *lef* and *pagA* with the BAPA probe sequence (S2 Table). Various combinations of markers, including a notable combination of Ba-1, BAPA, *capB*, and *lef*, accounted for a significant portion of the positive samples (S2 Table). Further details on other markers and their combinations are provided in S2 Table.

Microscopic evaluation of the 1708 blood smears detected 24.9% (425) of positive samples based on presence of bamboo-shaped, square-ended bacilli. However, when combining molecular and microscopy results, BAPA + *lef* + microscopy yielded 395 positives (23.1% of 1708 samples), Ba-1 + BAPA + microscopy had 398 positives (23.3% of 1708 samples), BAPA + *capB* + microscopy had 400 positives (23.4% of 1708 samples), *lef* + *capB* + microscopy had 401 positives (23.4% of 1708 samples), Ba-1 + *capB* + microscopy had 397 positives (23.2% of 1708 samples), Ba-1 + BAPA + *lef* + microscopy had 393 positives (23.00% of 1708 samples), while Ba-1 + BAPA *+ lef* + *capB* + microscopy yielded 391 positives (22.9% of 1708 samples).

There was a significant and moderate agreement between the binary outcomes of the molecular tests (combining Ba-1 + BAPA + *lef + capB*) and the results of the microscopic examination of the blood smears (kappa = 0.73, 95% CI: 0.67–0.78, *p*<0.0001). All samples were negative for the genomic island GI4 of Bcbva.

## Discussion

This study explored whether bacteria closely related to *B*. *anthracis* complicate anthrax surveillance and diagnostics using molecular markers from wildlife mortalities in KNP. The discovery of Bcbva, mobile genetic elements, and serological cross-reactions has highlighted the risk of misidentifying anthrax-causing bacteria. Molecular markers must therefore be carefully considered to avoid cross-reactions with closely related organisms in the same environment. Our analysis of the *gyrB* gene showed that blood smears can contain *Priestia* spp., *Peribacillus* spp. (both formerly *Bacillus* spp.), and *B*. *cereus sensu stricto*, which cross-react with common molecular markers like *pagA* (BAPA probe) or *lef* used in anthrax diagnostics [16,71]. These bacteria may be pathogenic, commensal, or contaminants. Using a combination of markers, as we did, reduces misidentification. Our findings showed good agreement between diagnoses based on microscopy and molecular techniques, suggesting these methods could accurately diagnose anthrax, potentially reducing reliance on culture confirmation. This shift could lower biosafety risks associated with traditional culture methods (safe disposal of enumerated spores), though further studies are needed to confirm these results.

The identification of species closely related to *B*. *anthracis* on diagnostic blood smears can complicate anthrax diagnosis as these species may share similar genetic markers with *B*. *anthracis*, leading to false positive results from molecular diagnostics. In addition, other genera, such as *Peribacillus* and *Priestia* [72–74], can also complicate anthrax diagnosis. These species may not share as many genetic markers with *B*. *anthracis* as the *B*. *cereus* group, but still have some similarities that could lead to false positive results as seen in this study when performing qPCR diagnostics using only *lef*, Ba-1, or *pagA* with BAPA probe sequence markers (Fig 5). For instance, *Peribacillus* and *Priestia* genera have been reported to have similar 16S rRNA gene sequences and protein profiles as *Bacillus* [75], which can lead to misidentification. This suggests the presence of other bacterial species that share similar *pagA* with BAPA probe sequence to that of *B*. *anthracis* with significant implications for *pagA*-based ELISA. The results of our study show that other closely related organisms can react to *pagA* with BAPA probe sequence and produce false positive results as hypothesised in a serological study conducted in KNP [19]. It is therefore necessary to consider using other genetic markers or a combination of markers to confirm the presence of *B*. *anthracis*.

Specifically, Lekota *et al*., [73] demonstrated that the genes for the capsular operons (*capABC*) are the ones that complicate anthrax diagnosis. Lekota *et al*., [73] reported that *capC* is not specific to *B*. *anthracis*. Thus, combining capsule markers such as the *capB* in this study with other markers increased the specificity. However, in this study, *capB* had a lower sensitivity (96.67%) that should be interpreted with caution owing to the small number of samples that were confirmed as *B*. *anthracis*. Because the virulence factors of *B*. *anthracis* occur in closely related *Bacillus* species [47], the combination of chromosome, toxin and capsule genes may yield the best diagnostic result as seen in this study. The BAPA + *lef* + Ba-1 combination showed 100% specificity, sensitivity, and accuracy.

Archival smears are a useful resource for retrospective studies and retrieval of environmentally persistent pathogens like *B*. *anthracis* [55]. We were only able to culture *B*. *anthracis*, as defined by microscopy, culture, molecular diagnosis, and sensitivity to penicillin and gammaphage, from four samples collected during the 2015 outbreak from impala (*Aepyceros melampus*). These samples did not include any of the 25 *B*. *anthracis* internal controls collected in 2012–2013. This indicates that the endospores were not viable after 10 years from these 25 smears, which were known to be *B*. *anthracis* cases from previous work. This agrees with the findings of Hassim [52] who reported that the longer a smear is stored, the harder it is to recover *B*. *anthracis*, and this may also affect the quality of the DNA extracted from such samples. Furthermore the 25 *B. anthracis* positive control isolates were isolated from follow-up collection of bone, hair, and tissue samples and not from the blood smears as sample type could influence isolation. The capsule found on the pXO2 plasmid was potentially missing for one of the 4 *B*. *anthracis* isolates obtained from the smears. This has previously been reported to occur in the long-term storage of isolates [76]. The mechanism of how the plasmids are lost is still not properly understood but it is hypothesised to be due to damage to the DNA or following nutrient deficiency over time [76]. This suggests the possibility that archival smears might benefit from storage in climate-controlled conditions to prolong their shelf life. Additionally, although *B*. *anthracis* can survive for extended periods, it is not a guarantee, indicating that storage conditions warrant further evaluation.

The chromosomal marker Ba-1 has been reported to be very specific to *B*. *anthracis* [41]. However, in this study, of the isolates that tested positive for Ba-1, only 4/42 were confirmed to be *B*. *anthracis* based on morphological, microscopic and sensitivity tests (gamma-phage and penicillin). The difference between our study and Zincke *et al*. [41] is likely due to the degradation of the samples in our study, which have been archived over time. It may also be due to the different samples pools, where Zincke *et al*. [41] evaluated the Ba-1 marker using samples of Bcbva, *B*. *cereus*, and *B*. *thuringiensis*, whereas the majority of the bacteria isolated in this study were *Priestia* spp, and *Peribacillus* spp. It is known that *Priestia* spp. and *Peribacillus* spp. are quite ubiquitous as they can be found in soil, faeces and the plant rhizospheres [70], complicating anthrax diagnosis. All samples in this study were negative for GI4, and there have been no reports of Bcbva outside of West Africa, suggesting it may not be present in KNP. Consequently, GI4 may not be a viable marker for pathogenic strains in southern Africa, although screening for Bcbva remains important since it could be overlooked in current diagnostic regimens. The ecological range of Bcbva, particularly in transitional areas between humid forests and dry savannas typical of *B*. *anthracis* habitats, is not fully understood. Investigating non-traditional regions using new diagnostic tools like Bcbva-specific proteins is crucial for understanding Bcbva’s distribution, assessing risks, and guiding future surveillance and research efforts. Developing geographic region-specific diagnostics could improve the identification of anthrax-like cases if such rare cases exist.

Accurate detection of *B*. *anthracis* can be enhanced by using a stepwise approach with multiple genetic markers, particularly when culture is not feasible. Studies by Blackburn *et al*. [44] and Zincke *et al*. [41] successfully employed Ba-1 in combination with MLVA-based or WGS-based methods to confirm species and prevent overestimation. In our study, relying on Ba-1 or *lef* markers alone produced non-specific results, but combining both markers reduced false positives, increasing specificity to 96.1%. More precise outcomes were achieved with combinations like BAPA + *capB*, Ba-1 + *capB*, and BAPA + *lef* + Ba-1, which showed high specificity and accuracy. However, these combinations risk misdiagnosing capsule-deficient *B*. *anthracis* isolates, as observed with AX2015-1136. The combination of BAPA + *lef* + Ba-1 proved to be the most reliable, achieving 100% specificity, sensitivity, and accuracy, making it a robust diagnostic strategy in the absence of culture and microscopy. Including *capB* is important for detecting capsule-producing *B*. *anthracis*, while MLVA and genotyping aid in identifying pXO2-positive samples and incorporating them into phylogenetic analyses, even when *capB* is absent [77,78].

The absence of other genetic markers and negative microscopic results suggest that *lef* is non-specific to *B*. *anthracis* and can be found in other species. In this study, *lef* appeared less specific than *pagA* with the BAPA probe or other markers used for anthrax diagnosis. This aligns with Zincke *et al*. [41], who demonstrated that *lef* could amplify *B*. *thuringiensis* serovar Kurstaki HD1 and *B*. *cereus* G9241, both of which carry a pXO1-like plasmid with anthrax toxin genes. Similarly, *lef* has been detected in non-*B*. *anthracis* pathogenic *B*. *cereus* in humans [79]. Incorporating multiple markers or techniques improves diagnostic accuracy, with marker combinations from both plasmids or plasmids and the chromosome reducing false positives. Adding microscopy further increased accuracy, minimizing variation in positive samples. The combination of genetic markers and microscopy can effectively diagnose *B*. *anthracis*, reducing reliance on culture. *Lef* was less specific than *pagA* and *capB*, with penicillin sensitivity noted in two non-*B*. *anthracis* isolates, while gamma phage sensitivity was exclusive to *B*. *anthracis*. Only *B*. *anthracis* isolates, except for AX2015-1136 (missing the capsule), tested positive for Ba-1, BAPA, *capB*, and *lef*. Effective diagnosis requires positive results for Ba-1 + BAPA + *lef* or combinations including *capB* (e.g., BAPA + *capB*, Ba-1 + *capB*, or BAPA + *lef* + *capB*).

The strong significant agreement between microscopic and molecular diagnosis in this study highlights the value of microscopy for onsite *B*. *anthracis* detection. Combining microscopy with qPCR from blood smear scrapings offers a significant advancement, potentially reducing reliance on traditional culture methods. This is particularly important amid rising bioterrorism threats, providing a rapid, specific, and safer alternative for identifying *B*. *anthracis* [80]. While microscopy can quickly identify *Bacillus* rods, it cannot offer a definitive diagnosis due to the presence of similar species, and its accuracy depends on the diagnostician’s expertise. Additionally, spore formation complicates both culture-based and molecular methods, requiring extra steps like heat or chemical treatment for germination [3,81]. qPCR, however, can directly target *B*. *anthracis* DNA, providing highly sensitive and specific identification. Using qPCR with blood smear scrapings bypasses the time-consuming culture process, enhancing diagnostic speed and safety [80], which is crucial for early response to anthrax outbreaks [82].

This study highlights the importance of not entirely replacing microscopy with molecular tests for diagnosing anthrax. While molecular techniques such as qPCR often demonstrate higher sensitivity, as shown in a study where qPCR outperformed microscopy in diagnosing cutaneous anthrax [83], microscopy remains a valuable tool. It provides critical insights into the clinical presentation and progression of the disease, especially in resource-limited or field settings. Previous studies have also emphasized the practicality of microscopy in such conditions [56], reinforcing its continued relevance alongside molecular diagnostics.

Combining different methods is especially important given recent reports of Bcbva possessing several characteristics of *B*. *anthracis* [12,15]. For example, organisms are non-hemolytic and both form rods in chains that can be difficult to differentiate. With advancements in next-generation sequencing and decreasing costs, leveraging computational methods with robust bioinformatics can significantly improve anthrax diagnosis and differentiation from Bcbva and other anthrax-like pathogens. This study’s findings have substantial implications for public health and One Health initiatives [84], contributing to more accurate, efficient, and accessible diagnostic approaches for anthrax detection, ultimately aiding in the prevention and control of the disease in livestock, wildlife, and human populations.

### Limitations of the study

Despite identifying *B*. *anthracis* in smears from the 2012–2015 outbreaks, culture success from these archived slides was limited to the 2015 outbreak, likely due to challenges in the viability of *B*. *anthracis* endospores in blood smears over time. The storage of the blood smears over an extended period may have impacted their quality and introduced possible bacterial containation. Additionally, the determination of sensitivity, specificity, and accuracy was based on only four positive samples and 25 internal controls. Therefore, assessing the performance of the assays on a larger number of samples, including more culture/gold standard-confirmed positive cases, would be beneficial.

## Conclusion

Results of this study demonstrate that diagnostic markers and techniques that are specific to *B*. *anthracis* could reduce the complications in detection that are currently experienced, especially with an increase in the exploration of the potential sharing of genetic material amongst the *B*. *cereus sensu lato* members. Microscopy remains a very valuable tool in confirming the presence of *B*. *anthracis* in the field and resource-limited settings, as well as a confirmatory tool. Accurate diagnosis with microscopy and combination of markers can reduce or eliminate the need for culture and bacterial proliferation. The presence of non-*B*. *anthracis* organisms harbouring similar genes may complicate anthrax diagnosis in the field. Lastly, the study identifies that the combination of Ba-1+BAPA+*lef* yields the most specific, sensitive, and accurate results. However, employing combinations such as BAPA+*lef*+*capB* along with microscopic analysis can enhance diagnostic confirmation, reduce false positives, and potentially minimize the need for culture, as revealed in this research. Nonetheless, it is important to note that the presence or absence of pXO2 is a crucial step in characterizing *B*. *anthracis*, especially for identifying true capsule-forming strains. Additionally, cultivation remains essential for collecting strains and extracting high-quality pure DNA for genetic analyses, such as whole genome sequencing, which is a reliable tool for differentiating different strains within the *B. cereus* group.

## Supporting information

S1 Fig Agarose gel image of *Bacillus anthracis* protective antigen gene region, *pagA* (BAPA), for *B. anthracis* and other bacterial species isolated from cultured blood smears obtained from wildlife mortalities in Kruger National Park, South Africa. The 100 bp (Thermo Scientific, USA) ladder was used. The *B*. *anthracis* Sterne and Vollum (labelled as *B*. *anthracis* V) strains served as the positive controls. *Bacillus cereus* ATCC3999 and distilled water (labelled as Negative) were used as negative controls. Sample numbers highlighted with blue rectangles indicate *B*. *anthracis* confirmed samples. The assay was repeated three times.
(TIF)

S1 Table Wildlife species in Kruger National Park, South Africa that tested positive for *Bacillus anthracis* protective antigen (*pagA* with BAPA probe sequence), lethal factor (*lef*), chromosomal marker (Ba-1) and the capsule region (*capB*) or a combination of these genetic markers and count of animals positive. (DOCX)

S2 Table Positive results of scraped blood smears using *Bacillus anthracis* protective antigen (BAPA), lethal factor (*lef*), chromosomal marker (Ba-1) and the capsule region (*capB*) molecular markers and marker combinations in probe-based real-time/quantitative polymerase chain reaction (qPCR), with "only" indicating exclusive positivity for the respective marker or combination. (DOCX)

S1 Text Confirmation of *pagA* Positivity in Blood Isolates through Conventional PCR. (DOCX)

S1 Dataset The S1 dataset encompasses various data used to generate the results in this article, including *Bacillus* species, Culture, PCR, Morphology, GTBR Report, Probe Smear PCR, Probe-only isolates, Smear Combined Count. (ZIP)
