# Supplementary figures and images for: Comparing microbiological and molecular diagnostic tools for the surveillance of anthrax
Source: PLoS Negl Trop Dis. 2024 Nov 21;18(11):e0012122. doi: 10.1371/journal.pntd.0012122 (PMC11620650; doi:10.1371/journal.pntd.0012122)

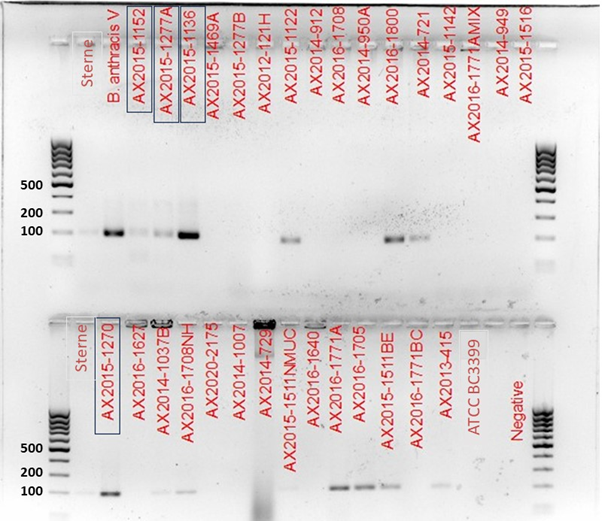

Supplement: S1 Fig — The 100 bp (Thermo Scientific, USA) ladder was used. The B. anthracis Sterne and Vollum (labelled as B. anthracis V) strains served as the positive controls. Bacillus cereus ATCC3999 and distilled water (labelled as Negative) were used as negative controls. Sample numbers highlighted with blue rectangles indicate B. anthracis confirmed samples. The assay was repeated three times. (TIF) [file pntd.0012122.s001.tif]
