# Supplementary material for: Comparing microbiological and molecular diagnostic tools for the surveillance of anthrax
Source: PLoS Negl Trop Dis. 2024 Nov 21;18(11):e0012122. doi: 10.1371/journal.pntd.0012122 (PMC11620650; doi:10.1371/journal.pntd.0012122)
Supplement: S1 Table — (DOCX) [file pntd.0012122.s002.docx]

Supplementary Table S1: Wildlife species in Kurger National Park, South Africa that tested positive for *Bacillus anthracis* protective antigen (*pagA* with BAPA probe sequence), lethal factor (*lef*), chromosomal marker (Ba-1) and the capsule region (*capB*) or a combination of these genetic markers and count of animals positive. Supplementary Table S1: Wildlife species in Kurger National Park, South Africa that tested positive for *Bacillus anthracis* protective antigen (*pagA* with BAPA probe sequence), lethal factor (*lef*), chromosomal marker (Ba-1) and the capsule region (*capB*) or a combination of these genetic markers and count of animals positive.

| Common name of species | Scientific name of species | Number examined | Percent and number (n) of smears positive for *B. anthracis by one or more markers* |
| --- | --- | --- | --- |
| African buffalo | *Syncerus caffer* | 288 | 38.2%(110) |
| African Elephant | *Loxodonta africana* | 142 | 36.0%(51) |
| African wild dog | *Lycaon pictus* | 1 | 0%(0) |
| Banana bat | *Musonycteris harrisoni* | 1 | 0%(0) |
| Black-backed jackal | *Lupulella mesomelas* | 2 | 0%(0) |
| Blue wildebeest | *Connochaetes taurinus* | 20 | 20.0%(4) |
| Bushbuck | *Tragelaphus scriptus* | 6 | 50.0%(3) |
| Brown hyena | *Hyaena brunnea* | 1 | 0%(0) |
| Chacma Baboon | *Papio ursinus* | 11 | 81.9%(9) |
| Cheetah | *Acinonyx jubatus* | 2 | 0%(0) |
| Common duiker | *Sylvicapra grimmia* | 1 | 0%(0) |
| Common eland | *Taurotragus oryx* | 4 | 25.0%(1) |
| Giraffe | *Giraffa camelopardalis* | 32 | 25.0%(8) |
| Hippopotamus | *Hippopotamus amphibius* | 49 | 34.7%(17) |
| Impala | *Aepyceros melampus* | 525 | 70.3%(369) |
| Greater kudu | *Tragelaphus strepsiceros* | 120 | 76.7%(92) |
| Large-spotted genet | *Genetta tigrina* | 1 | 100%(1) |
| Leopard | *Panthera pardus* | 2 | 0%(0) |
| Lion | *Panthera leo* | 2 | 0%(0) |
| Nile crocodile | *Crocodylus niloticus* | 1 | 100%(1) |
| Nyala | *Tragelaphus angasii* | 47 | 44.7%(21) |
| Ostrich | *Struthio camelus* | 3 | 0%(0) |
| Roan antelope | *Hippotragus equinus* | 29 | 72.5%(21) |
| Spotted hyena | *Crocuta crocuta* | 16 | 0%(0) |
| Steenbok | *Raphicerus campestris* | 11 | 27.3%(3) |
| Common tsessebe | *Damaliscus lunatus* | 4 | 25.0%(1) |
| Vervet monkey | *Chlorocebus pygerythrus* | 4 | 50.0%(2) |
| Warthog | *Phacochoerus africanus* | 10 | 40.0%(4) |
| Waterbuck | *Kobus ellipsiprymnus* | 22 | 41.0%(9) |
| White rhinoceros | *Ceratotherium simum* | 114 | 18.5%(21) |
| Not labelled |  | 150 | 64.0%(96) |
|  | **Total** | **1708** | **890** |
