# Supplementary material for: Comparing microbiological and molecular diagnostic tools for the surveillance of anthrax
Source: PLoS Negl Trop Dis. 2024 Nov 21;18(11):e0012122. doi: 10.1371/journal.pntd.0012122 (PMC11620650; doi:10.1371/journal.pntd.0012122)
Supplement: S2 Table — (DOCX) [file pntd.0012122.s003.docx]

The DNA directly extracted from blood smears (n = 1708 samples) revealed 890 samples that tested positive for at least one of the molecular markers evaluated (Table S1). However, of these 890 samples, 165 (18.5%, Table S2) tested positive for *lef* only and 112 (12.6%, Table S2) for *pagA* with BAPA probe sequence only, and not for any other marker. Details of other markers and combinations of markers are found in Table S2. A combination of Ba-1 + BAPA + *cap*B + *lef* yielded a total of 393 positive samples (44.2% of the 890 samples; Table S2).

**Table S2:** Positive results of scraped blood smears using *Bacillus anthracis* protective antigen (BAPA), lethal factor (*lef*), chromosomal marker (Ba-1) and the capsule region (*capB*) molecular markers and marker combinations in probe-based real-time/quantitative polymerase chain reaction (qPCR), with "only" indicating exclusive positivity for the respective marker or combination.

| **Molecular Markers** | **Count (%)** |
| --- | --- |
| Ba-1 only | 13 (1.5) |
| *lef* only | 165 (18.5) |
| BAPA only | 112(12.6) |
| capB only | 5 (0.6) |
| Ba-1+*lef* only | 13(1.5) |
| Ba-1+capB only | 3(0.3) |
| Ba-1 + BAPA only | 45(5.1) |
| BAPA + *lef* only | 131(14.7) |
| BAPA + capB only | 10(1.1) |
| Ba-1+BAPA+capB+*lef* | 393 (44.2) |
