# Supplementary material for: Comparing microbiological and molecular diagnostic tools for the surveillance of anthrax
Source: PLoS Negl Trop Dis. 2024 Nov 21;18(11):e0012122. doi: 10.1371/journal.pntd.0012122 (PMC11620650; doi:10.1371/journal.pntd.0012122)
Supplement: S1 Text — (DOCX) [file pntd.0012122.s004.docx]

**Supplementary Information**

**Comparing microbiological and molecular diagnostic tools for the surveillance of anthrax.**

Sunday O. Ochai^1,2*^, Ayesha Hassim^1^, Edgar H. Dekker^3^, Thuto Magome^4^, Kgaugelo E. Lekota^4^, S. Marcus Makgabo^1,5^, Lin‑Mari de Klerk‑Loris^2^, O. Louis van Schalkwyk^1,3,6^ Pauline L. Kamath^7^ , Wendy C. Turner^8^, Henriette van Heerden^1^

^1^ Department of Veterinary Tropical Diseases, Faculty of Veterinary Science, University of Pretoria, Onderstepoort, South Africa.

^2^Antimicrobial Research Unit, College of Health Sciences, University of KwaZulu-Natal, Private Bag X54001, Durban, 4000, South Africa

^3^ Office of the State Veterinarian, Department of Agriculture, Forestry and Fisheries, Government of South Africa, Skukuza, South Africa

^4^ Unit for Environmental Sciences and Management, Microbiology, North West University, Potchefstroom, South Africa

^5^ Department of Life and Consumer Sciences, College of Agriculture and Environmental Sciences, University of South Africa (UNISA), Florida Campus, Roodepoort 1709, South Africa.

^6^ Department of Migration, Max Planck Institute of Animal Behavior, Radolfzell, Germany

^7^ School of Food and Agriculture, University of Maine, Orono, Maine, U.S.A.

^8^ U.S. Geological Survey, Wisconsin Cooperative Wildlife Research Unit, Department of Forest and Wildlife Ecology, University of Wisconsin-Madison, Madison, Wisconsin, U.S.A

* Correspondence: Sunday O. Ochai; [s.o.ochaijr@gmail.com](mailto:s.o.ochaijr@gmail.com)

Any use of trade, firm, or product names is for descriptive purposes only and does not imply endorsement by the U.S. Government.Supplementary methodology:

**Conventional PCR**

The 14 isolates from cultured blood smears that tested positive for *pagA* with BAPA probe seuence by the two qPCR methods described above were subjected to conventional PCR to allow for gel visualisation as described by Lekota et al. (2016) [42]. The reaction was set up with a total volume of 10 μL, containing 1x MyTaq PCR Master Mix (Bioline, Taunton, USA), 2 mM MgCl2, 0.2 µM of each *pagA* primer (Table 1), and 2 ng of the target DNA. The PCR amplification began with an initial denaturation step at 94°C for 5 min, followed by 35 cycles of denaturation at 94°C for 30 sec, annealing at 55°C for 30 sec. and extension at 72°C for 30 seconds, concluding with a final extension at 72°C for 5 min. The *B. anthracis* Vollum strain served as a positive control. *Bacillus cereus* ATCC3999 and distilled water served as negative controls, ensuring the assay's specificity. Post-amplification, the PCR products were subjected to electrophoresis on a 3% ethidium bromide-stained agarose gel at 100 V for 90 min and visualized under UV light to confirm the presence of *Bacillus anthracis*-specific amplicons.

Supplementary results

The 14 samples that tested positive for pagA using qPCR were further confirmed with conventional PCR, as illustrated in Figure S1. All 14 samples maintained their positive status upon confirmation with conventional PCR, reinforcing the reliability of the qPCR results.
